# Pharmacist-led iodinated contrast media infusion risk assessment service

**DOI:** 10.3389/fphar.2023.1161621

**Published:** 2023-05-09

**Authors:** Huiyan Jiang, Yuan Li, Xiaoyan Wu, Hongming Yu, Xin Zhang, Weihong Ge, Simin Yan

**Affiliations:** ^1^ Department of Pharmacy, Nanjing Drum Tower Hospital, Affiliated Hospital of Medical School, Nanjing University, Nanjing, Jiangsu, China; ^2^ Department of Radiology, Nanjing Drum Tower Hospital, Affiliated Hospital of Medical School, Nanjing University, Nanjing, Jiangsu, China

**Keywords:** iodinated contrast media, pharmacist, multidisciplinary, risk assessment, contrast-enhanced computed tomography

## Abstract

**Background:** With the increasing development of medical imaging, the use of iodinated contrast media has become more widespread. Adverse reactions caused by iodinated contrast media have drawn much attention. Despite this, there is still a lack of unified standards for the safe infusion process of iodinated contrast media in clinical practice both domestically and internationally.

**Objectives:** Establishing a risk management service system to better predict the risks associated with iodinated contrast media infusion, reduce the incidence of adverse reactions and minimize patient harm.

**Method:** A prospective interventional study was carried out from April 2021 to December 2021 at Nanjing Drum Tower Hospital in China. During this study, a service system was established to manage the risks associated with the infusion of iodinated contrast media. Personalized risk identification and assessment were performed by a pharmacist-led multidisciplinary team before iodinated contrast media infusion. Early warning, prevention, and adverse reaction management were performed according to different risk levels during and after infusion.

**Results:** A multidisciplinary team led by pharmacists was established to evaluate the risks associated with infusion of iodinated contrast media. A total of 157 patients with risk factors related to the iodinated contrast media were screened out, which prevented 22 serious adverse events and enhanced the quality of medical care. All participants expressed high satisfaction with the service.

**Conclusion:** Through practical exploration, the pharmacist-led multidisciplinary team can provide advance warning and effectively limit the risks of adverse reactions caused by iodinated contrast media to a preventable and controllable level. This approach serves as a valuable reference for developing strategies and schemes to reduce the incidence of such reactions. Therefore, we encourage the implementation of this intervention in other areas of China.

## 1 Introduction

With the advancements in medical imaging, it is estimated that over 100 million procedures are performed worldwide each year using iodinated contrast media (ICM) (Böhm et al., 2017). Despite the improvement in the properties of ICM, national monitoring data and literature indicate that the growing usage has resulted in an increase in adverse drug reactions (ADR) (Chen et al., 2014). Consequently, the safety of ICM has become a matter of increasing concern.

Contrast-associated acute kidney injury (CA-AKI) is a sudden drop in kidney function within 48 h of receiving intravascular Iodinated Contrast Media (ICM). It has become the third most common cause of hospital-acquired acute kidney injury (AKI) (Nash et al., 2002). The occurrence of CA-AKI in the general population is low, ranging from 1% to 2% (Everson et al., 2020). However, patients with risk factors have a significantly higher incidence, ranging from 5% to 30% (Davenport and Perazella, 2020). Kidney damage can have serious consequences, such as an increased risk of complications during hospitalization, including the development of chronic kidney disease (CKD) (1%), requiring renal replacement therapy (0.06%), or even death (Kooiman et al., 2012). Hypersensitivity reaction to ICM is another common problem that causes concern among healthcare workers. The rate of true anaphylaxis is reported to be between 0.3% and 3% (Chen et al., 2017; Topaz et al., 2018), whereas severe reactions occur in 0.03%–1.6% of injections (Newmark et al., 2012). The most common reaction is an itchy rash all over the body or locally. In severe cases, patients may develop diffuse facial and laryngeal edema, bronchospasm, and dyspnea, leading to anaphylactic shock, which can result in permanent illness or even death if not managed properly. In addition, the use of ICM can affect the progression of certain diseases. In patients with hyperthyroidism, the use of ICM can worsen their condition and even lead to a thyroid crisis (Chen et al., 2014). ICM injections can also cause significant hemodynamic changes in patients with valvular heart disease and pulmonary hypertension (John and Yadav, 2019). Moreover, ICM can worsen myasthenia symptoms (Somashekar et al., 2013) and cause a hypertensive crisis in patients with pheochromocytoma (Nakano et al., 2011).

Current consensus and guidelines provide information on the types of adverse reactions common to ICM and their associated risk factors, highlighting the importance of risk assessment (Li et al., 2018; van der Molen et al., 2018; Davenport et al., 2020; ACR comittee on drugs and contrast media, 2022). However, through the preliminary survey, our hospital still faces the following challenges: 1) Some patients have complex medical histories that may increase their risk of adverse reactions to ICM infusion. 2) Currently, nurses in our hospital are only responsible for basic inquiries before administering ICM, and there is a lack of a complete and standardized risk assessment and intervention system to predict and address adverse reactions promptly. 3) There is a shortage of qualified pharmacists to determine whether medications taken by patients during screening may increase their risk of ADR. 4) A standardized risk assessment workflow has not been established, and communication among doctors, nurses and pharmacists, regarding their respective roles and responsibilities is unclear. 5) In some cases, the risk of ADR may be overestimated, leading to delayed diagnosis and treatment.

The key step to ensure a smooth imaging examination is the infusion of ICM. To achieve an efficient, reasonable, safe and standardized infusion process, the medical technicians must cooperate and maintain a high level of performance. Additionally, the pharmacist plays a crucial role in a multidisciplinary team responsible for managing adverse drug events in hospitals, promoting the safe use of drugs (Choukroun et al., 2021; Shrestha et al., 2022). Therefore, in April 2021, a multidisciplinary collaborative management team for risk assessment of ICM infusion was established in our hospital, consisting of clinicians, nurses and pharmacists. Stratified assessment and personalized management of risk were implemented throughout the entire process, from the patients’ appointment to the injection of ICM.

### 1.1 Aim of the study

The study aimed to develop a risk management service system that enhances the ability to predict and mitigate the risks associated with the infusion of ICM, thereby reducing the incidence of adverse reactions and improving drug safety in patients.

## 2 Method

### 2.1 Study participants

**FIGURE 1:**
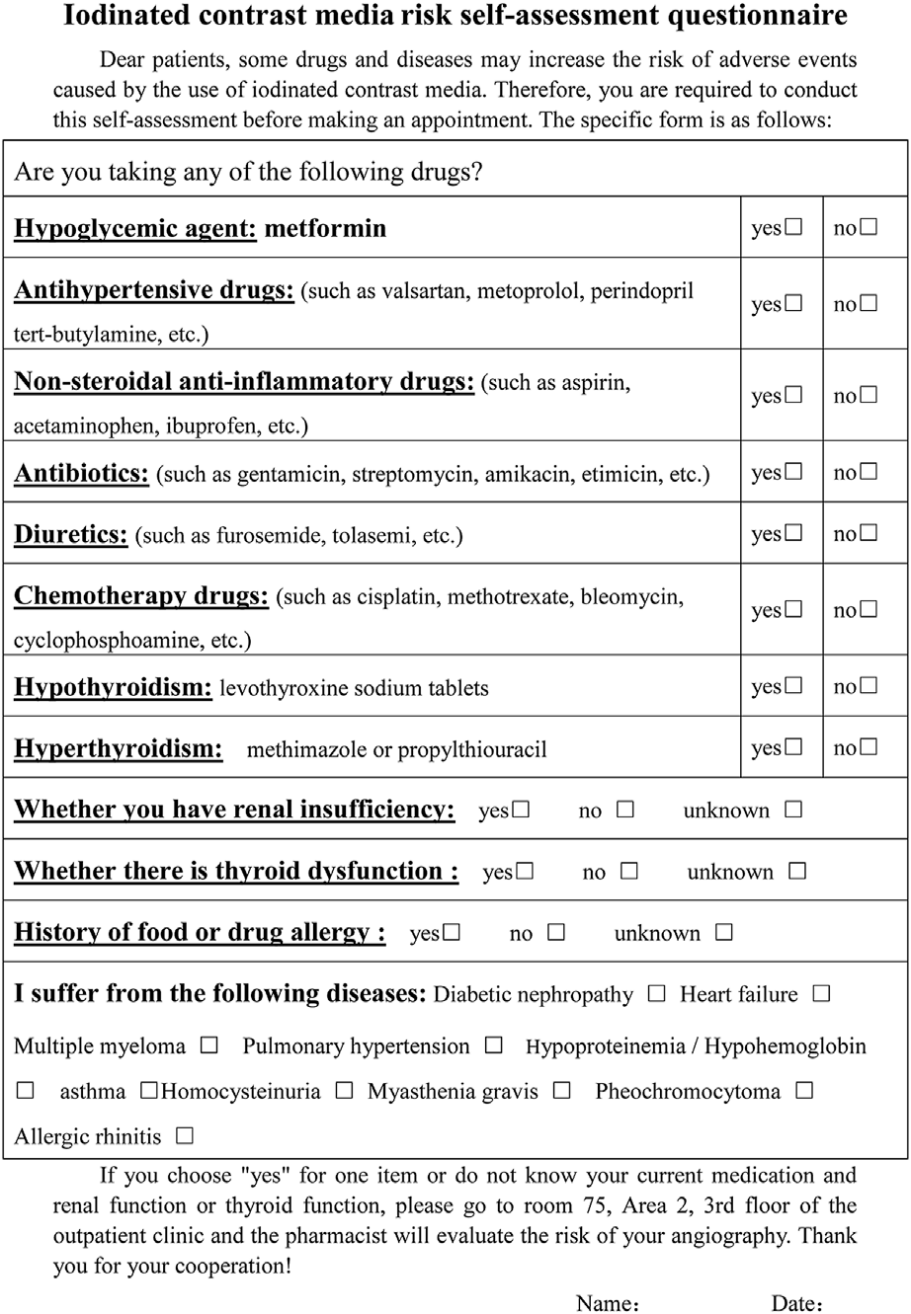
Iodinated contrast media risk self-assessment questionnaire.

The risk early warning and prevention study was conducted at Nanjing Drum Tower Hospital from April 2021 to December 2021. A risk self-assessment questionnaire was designed for patients based on the main risk factors associated with ICM by referring to relevant literature and guides (Chen et al., 2014; Rosado Ingelmo et al., 2016; van der Molen et al., 2018; John and Yadav, 2019; Davenport et al., 2020; ACR comittee on drugs and contrast media, 2022) (Figure 1). Inclusion criteria: 1) patients who underwent routine contrast-enhanced computed tomography (CECT) from April 2021 to December 2021; 2) Volunteer to participate in this risk assessment service; 3) patients were able to maintain good communication with researchers to complete this study as required; 4) there was no age limit for patients. Patients who were pregnant or scheduled for surgery within 1 week were excluded from the study. Patients who agreed to participate signed an informed consent form and arranged meetings with pharmacists after completing the self-assessment questionnaire.

### 2.2 Building a multidisciplinary team

A comprehensive ICM infusion risk management service was carried out by a pharmacist-led, multidisciplinary team. Clinical pharmacists who participated in this study were pharmacy graduate students with at least 5 years of clinical experience and had successfully completed the Medication Therapy Management Program offered by the University of Minnesota College of Pharmacy. All team members received uniform training before providing this service. Besides, they are responsible for understanding the types and uses of contrast media, as well as being familiar with their potential adverse reactions, identifying corresponding risk factors, and knowing how to prevent and treat adverse reactions correctly. The roles and responsibilities of the multidisciplinary team members are as follows: Clinical departments: 1) Diagnosis of patients’ condition and ordered a CECT scan; 2) Balance the potential risks of contrast media administration with diagnostic benefits and consider alternative plans when the risks are large. Radiologists: 1) Preparing contrast media and high-pressure syringe pipeline; 2) Communicating with patients and take appropriate posture; 3) Set dose and flow rate; 4) Watching closely for adverse reactions during the CECT examination. Pharmacists: 1) Responsible for stratified risk assessment before infusion of ICM. 2) Related risk explanation and adequate hydration guidance; 3) Developing strategies for managing adverse reactions and informing nurses of its use 4) follow-up of patients. Radiology nurses: 1) Checking the patients’ basic information 2) Confirming whether there are contraindications for examination again 3) Evaluating the skin and blood vessels at the puncture site, placing indwelling needle. 4) Telling patients to stay under observation, quickly identifying adverse reactions and giving timely and appropriate treatment according to the patient’s condition after examination, extracting needle correctly. In addition, a staff member was set up to book examination time and guide patients to fill in the risk self-assessment form.

### 2.3 Establish risk assessment workflow

**FIGURE 2:**
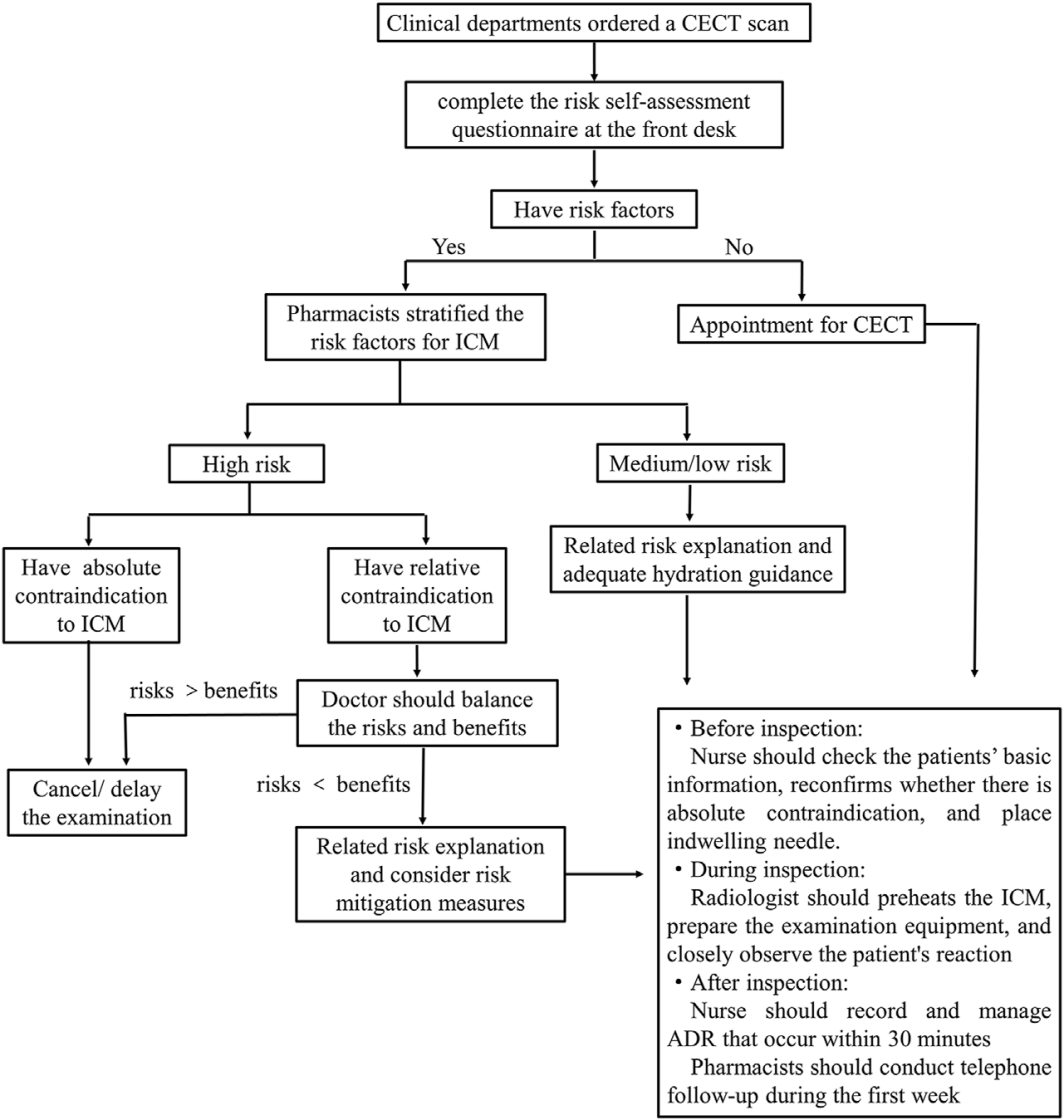
Iodinated contrast media infusion risk assessment flowchart in our hospital. CECT, contrast-enhanced computed tomography; ICM, iodinated contrast media; ADR, adverse drug reactions.

ICM infusion risk assessment flowchart was established in our hospital (Figure 2). After the doctor ordered a CECT scan, the front desk personnel guided the patients to fill in the risk self-assessment form before the appointment. When patients have contrast risk factors, a comprehensive risk assessment should be conducted face-to-face with a pharmacist. Pharmacist classified patients into high, medium and low risk from three aspects: anaphylaxis, CA-AKI and others. Examination were cancelled or delayed if there were absolute contraindications. If there were relative contraindications, the doctor balanced the potential risks of contrast media administration with diagnostic benefits and considered alternative plans or canceled the examination. All patients were informed by pharmacists of the risks and precautions associated with the ICM, and monitored throughout the examination process by members of the multidisciplinary team. All the ADRs were recorded within half an hour and a week after examination.

### 2.4 Risk stratification assessment criteria

**FIGURE 3:**
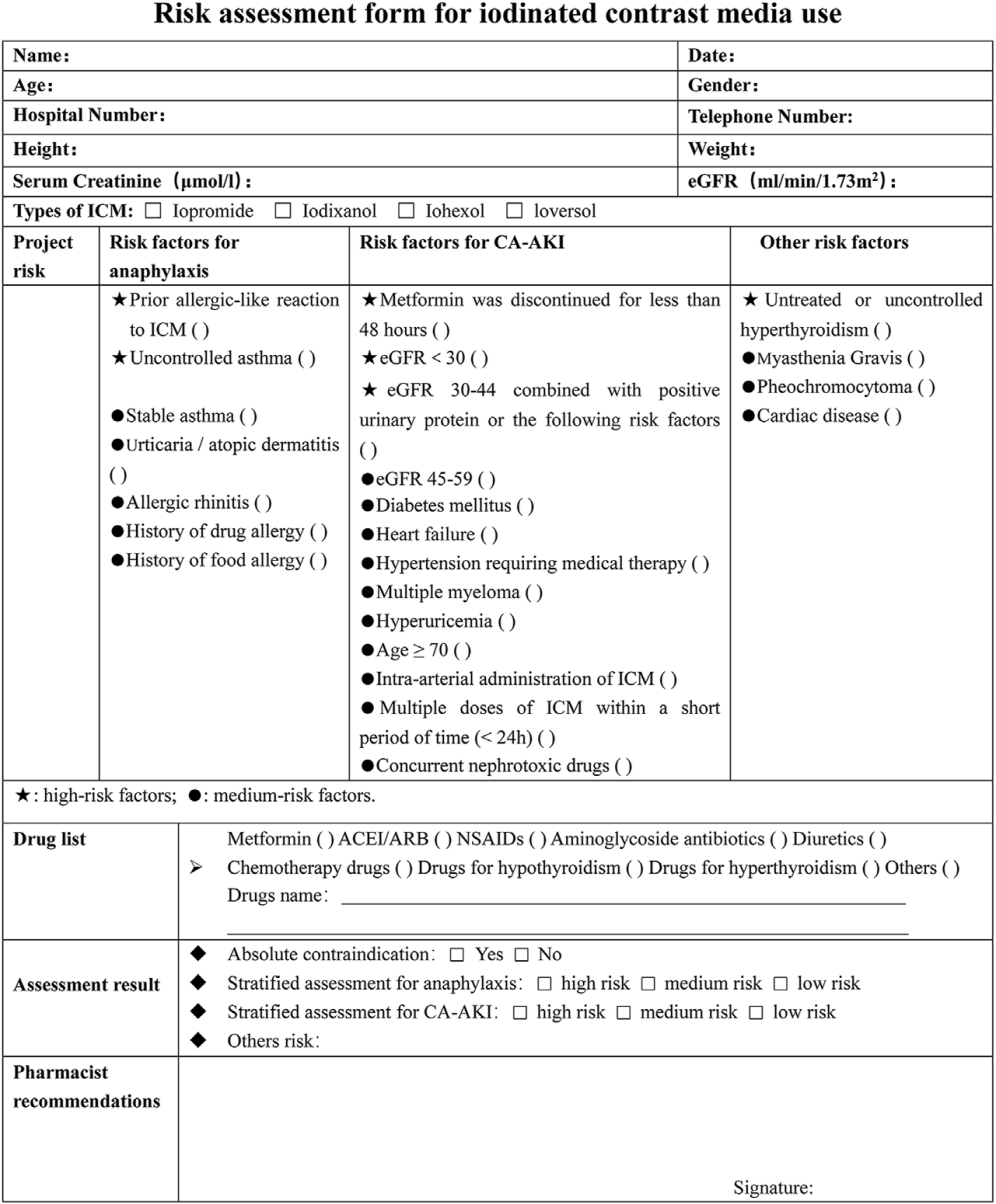
Risk assessment form for iodinated contrast media use. ICM, iodinated contrast media; eGFR, epidermal growth factor receptor; NSAIDs, nonsteroidal anti-inflammatory drugs; ACEI/ARB, angiotensin-converting enzyme inhibitor/angiotensin receptor blocker; CA-AKI, contrast-associated acute kidney injury.

The risk-stratified assessment form was designed by referring to a large number of guidelines and literatures (Figure 3). Each risk factor related to the use of ICM was carefully reviewed and sorted into one of three categories: high risk, medium risk, or low risk. The table identifies high-risk factors with ★ and medium-risk factors with a ●. At our hospital, patients with uncontrolled asthma, untreated or uncontrolled hyperthyroidism, or who have not discontinued metformin for at least 48 h are considered absolute contraindications for ICM. For patients who have experienced allergic-like reactions in the past, doctors will evaluate the risks and benefits of using a different type of ICM or canceling the examination altogether. If a patient has at least one high-risk factor, they are classified as high risk, regardless of whether they also have moderate-risk factors. If a patient has only one or more moderate-risk factors and no high-risk factors, they are classified as medium risk. Patients with neither high-risk nor moderate-risk factors are considered low risk.

### 2.5 Management of ADRs by ICM in our hospital

Based on established guidelines, we developed treatment standards for managing the common adverse reactions of ICM (Chen et al., 2014; Li et al., 2018; ACR comittee on drugs and contrast media, 2022). All patients were closely monitored for at least 30 min after the examination. Mild allergic reactions such as itching and hives were carefully observed, and moderate reactions were typically treated with anti-H1 blockers. Severe allergic reactions require treatment with epinephrine, while oxygen and inhaled β2-agonists may be administered to alleviate bronchospasm. High-risk patients for CA-AKI (contrast-induced acute kidney injury) received typical oral hydration volume expansion therapy, and renal function was reevaluated 48 h after the examination (Sebastià et al., 2021). In the event of hypertensive crises due to pheochromocytoma, phentolamine was administered. For the treatment of venous air embolism, patients were given 100% oxygen and positioned on their left side. If ICM extravasates (leaks out of the vein), the affected limb should be elevated above the level of the heart.

### 2.6 Patient satisfaction surveys

Each participant was given a four-question survey to assess patient satisfaction (refer to Table 1). Each answer was rated on a scale of 1–5, with 1 being the worst and 5 being the best response. The total score, which ranged from 4 to 20, was then categorized into three groups: low satisfaction (4–9), moderate satisfaction (10–14), and high satisfaction (15–20).

**TABLE 1:** Satisfaction survey.

Questions
1. The service enhanced my adherence to the risks of iodinated contrast media infusion
2. When I finished the assessment, I clearly understood the risks to be aware of during the inspection
3. The expertise of the multidisciplinary service team
4. I recommend the service to be continued

### 2.7 Statistical analysis

A Microsoft Access database was designed to facilitate data collection. Completed questionnaires were analyzed by the Statistical Package for the Social Sciences software version 26 (SPSS, International Business Machines Corp., Armonk, New York, United States). Ratios were used for categorical variables, and mean ± standard deviation (SD) for continuous variables of descriptive data.

## 3 Results

The study enrolled a total of 157 patients, of whom 61.1% were female. Details on patients’ demographics and other clinical characteristics are given in Table 2. According to the risk stratification criteria, 22 patients (14.0%) were classified as high risk, 125 patients (79.6%) as moderate risk, and 10 patients (6.4%) as low risk. Among patients at high risk, 16 patients had a prior allergic-like reaction to ICM, 1 had uncontrolled asthma, 1 was taking metformin, 2 had eGFR of 30–44 mL/min/1.73m^2^ combined with positive urinary protein or others CA-AKI risk factors, and 2 had untreated or uncontrolled hyperthyroidism. Out of these 17 patients (10.8%) at high risk of anaphylaxis, 12 attempted to switch to ICM with different ingredients, 1 patient chose magnetic resonance imaging instead of CECT scanning, 3 patients canceled the examination, and the patient with uncontrolled asthma delayed the examination. Due to the necessity of diagnosis, patients at high risk of CA-AKI were usually examined after receiving adequate hydration. Additionally, patients with untreated or uncontrolled hyperthyroidism were advised to visit an endocrinologist and delay the examination. It was further discovered that when patients with no history of ICM use were first exposed to ICM, potential risk factors were easily underestimated or ignored due to a lack of self-knowledge. Effective communication of drug risks significantly improved patients’ knowledge about the risks of ICM.

**TABLE 2:** Demograghic and general clinical characteristics of the patients (*n* = 157^a^).

Demograghic and general clinical characteristics	*n* (%)
Age (years); mean ± SD	54 ± 16
**Gender**	
Male	61 (38.9)
Female	96 (61.1)
**Types of ICM**	
Iohexol Injection (Omnipaque)	52 (33.1)
Iodixanol Injection (Visipaque)	34 (21.7)
Iopromide Injection (Ultravist)	71 (45.2)
**Patients at anaphylaxis risk**	
Prior allergic-like reaction to ICM	16 (10.2)
Uncontrolled asthma	1 (0.6)
Stable asthma	7 (4.5)
Urticaria/atopic dermatitis	15 (9.6)
Allergic rhinitis	9 (5.7)
History of drug allergy (ICM was not included)	100 (63.7)
History of food allergy	7 (4.5)
Had two or more anaphylaxis risk factors	17 (10.8)
**Patients at CA-AKI risk**	
Metformin was discontinued for less than 48 h	1 (0.6)
eGFR less than 30 mL/min/1.73m^2^	0 (0)
eGFR of 30–44 mL/min/1.73m^2^	3 (1.9)^b^
eGFR of 45–59 mL/min/1.73m^2^	5 (3.2)
eGFR of 60–89 mL/min/1.73m^2^	10 (6.4)
Diabetes mellitus	4 (2.5)
Heart failure	0 (0)
Hypertension requiring medical therapy	16 (10.2)
Multiple myeloma	0 (0)
Hyperuricemia	7 (4.5)
Age ≥70	27 (17.2)
Intra-arterial administration of ICM	31 (19.7)
Multiple doses of ICM within a short period of time (<24 h)	1 (0.6)
Concurrent nephrotoxic drugs	11 (7.0)
Had two or more CA-AKI risk factors	21 (13.4)
**Patients with thyroid dysfunction**	
Untreated or uncontrolled hyperthyroidism	2 (1.3)
Controlled hyperthyroidism	3 (1.9)
Subclinical hyperthyroidism	1 (0.6)
Hypothyroidism	5 (3.2)
Others	5 (3.2)

SD, standard deviation.

^a^ As multiple risk factors were compounded, the numbers do not equal 157.

^b^ There were 2 patients with eGFR of 30–44 mL/min/1.73 m^2^ combined with positive urinary protein or others CA-AKI risk factors; ICM, iodinated contrast media; eGFR, epidermal growth factor receptor; CA-AKI, contrast-associated acute kidney injury.

Patients who have risk factors are more likely to experience adverse reactions to contrast media. Our study found that 29 cases (18.4%) experienced allergic-like or physiological reactions to ICM. Most of these reactions were mild and included localized hives, itching, coughing, fever, dizziness, and wheezing. 7 cases (4.5%) experienced moderate adverse reactions within 48 h of the scan, including widespread hives, itching, nausea, palpitations, and mild wheezing. Patients with significant symptoms were treated with anti-H_1_ blockers, and all improved within a week. The study found no significant difference in the levels of serum creatinine or thyrotropin in patients with kidney or thyroid problems before and after the scan. None of the patients experienced severe adverse reactions related to the contrast media within the first week after the scan. All patients expressed high levels of satisfaction with the pharmacist-led multidisciplinary risk assessment service for contrast media infusion, and the recommendation was to continue using this service.

## 4 Discussion

### 4.1 Statement of key findings

Clinicians and radiologists face confusion when assessing patients due to the numerous risk factors outlined in various guidelines. Currently, there are no agreed-upon evaluation criteria to compare the risks and benefits of using ICM in clinical practice. Due to the unclear risk factors, most Chinese hospitals, particularly those in county areas, often choose to forgo examinations to avoid the risk of adverse reactions, which can complicate diagnoses (Li et al., 2016). The level of risk associated with ICM usage has become a significant concern. In our study, we have developed a comprehensive multidisciplinary risk assessment and risk management program for patients using ICM. All patients are required to undergo a risk assessment, which includes three areas of risk classification (anaphylaxis, contrast-induced acute kidney injury (CA-AKI), and other risks), three categories of risk (high, medium, and low), and three options following risk identification (elimination, reduction, or acceptance). The likelihood of developing risk increases exponentially with cumulative risk factors. Risk stratification tools help determine the level of risk for individual patients, allowing those at low risk to avoid unnecessary tests or overly burdensome preventive treatments, while those at high risk can receive effective preventive measures with close supervision. Our ultimate goal is to provide patients with personalized risk prevention and response programs.

Current evidence suggests that food and drug allergies are common, and have been increasing in prevalence in the last few decades (Sousa-Pinto et al., 2017; Loh and Tang, 2018). In our study, we found that 86.6% of patients had a history of allergy, atopy, or asthma, and many of them had multiple drug and food allergies or were allergic to two or more substances. The primary reason behind repeated allergic-like reactions was a prior allergic-like reaction to ICM (ACR comittee on drugs and contrast media, 2022). Our findings suggest that high-risk patients who had a previous allergic reaction without prophylactic medication had a 17.6% (3 of 12 examinations) chance of experiencing repeated allergies, which is similar to previous reports (Meth and Maibach, 2006; Davenport et al., 2009; Doña et al., 2020). Although the severity may not be that serious, patients with atopic diseases such as asthma or allergic predisposition are at increased risk of allergic reactions to ICM. The relative risk of an allergy-type reaction to ICM in people with a history of systemic allergies to several substances (excluding ICM) is twice as high as in the general population (Schopp et al., 2013).

Patients with asthma are nearly five times more likely to develop bronchospasm (Morcos, 2005). Therefore, it is crucial to consider any history of allergy-type reactions to ICM, as well as multiple allergies or asthma when evaluating patients for their risk of allergic reactions to ICM. While premedication (corticosteroid and antihistamine) can help prevent a recurrence of hypersensitivity reactions to contrast media, breakthrough reactions are still possible. About 10% of people still experience a breakthrough reaction while taking steroids, especially if the previous reaction was severe (Davenport et al., 2009). Recent research suggests that switching to a non-allergenic contrast media is more effective in reducing subsequent reactions than steroid premedication (Schrijvers et al., 2019; McDonald and Larson, 2021). However, ICM use is unavoidable in many disease evaluations, so for high-risk patients, we preferred to change the type of ICM.

Contrast-induced acute kidney injury (CI-AKI) refers to the rapid deterioration of renal function shortly after the administration of ICM. CI-AKI is a type of CA-AKI, which is associated with the use of contrast medias. However, there are few studies that distinguish CI-AKI from other types of CA-AKI in a suitable control group (ACR comittee on drugs and contrast media, 2022). Therefore, the more inclusive term CA-AKI is often used. Instead of serum creatinine-based screening, it is recommended to use epidermal growth factor receptor (eGFR) screening to identify individuals who may be at risk of CA-AK (Nyman et al., 2018; Davenport et al., 2020; ACR comittee on drugs and contrast media, 2022). The primary risk factor for CA-AKI is pre-existing renal impairment, particularly in patients with eGFR<30 min^−1^ 1.73^–2^, who have a 30% risk of developing CA-AKI, much higher than the general population (Davenport et al., 2020). Patients with a personal history of kidney disease (e.g., chronic kidney disease, solitary kidney, kidney cancer, kidney surgery, remote AKI, kidney ablation, albuminuria) should undergo renal function determination before undergoing any examination that involves the use of contrast medias. Other risk factors for CA-AKI include the use of nephrotoxic drugs, high doses of ICM within a short period of time (<24 h), multiple myeloma, hyperuricemia (serum uric acid level >7 mg/dL in men and >6 mg/dL in women), intra-arterial administration of ICM, advanced age, and diabetes mellitus (Schönenberger et al., 2019; Davenport et al., 2020; Everson et al., 2020; ACR comittee on drugs and contrast media, 2022). When administering concurrent nephrotoxic drugs (e.g., anti-inflammatories, antibiotics such as vancomycin and aminoglycosides, chemotherapeutic agents such as cisplatin and methotrexate, diuretics, cyclosporine A, and others), doctors should carefully monitor their patients’ renal function and hydration levels (ACR comittee on drugs and contrast media, 2022). The risk of CA-AKI increases exponentially with the presence of multiple risk factors. Risk stratification tools can help determine the magnitude of risk for CA-AKI, allowing low-risk individuals to avoid unnecessary prophylactic treatments and kidney function testing. Perioperative renal protection measures can benefit patients who are at high risk. However, there are some limitations to clinical prediction rules, which hinder their application in daily practice. For instance, the predictive power of these models for CA-AKI ranges from poor to excellent, and none of the models have been evaluated in clinical practice. Additionally, some model characteristics include contrast volume, which is usually not known until the imaging examination is complete.

The majority of current risk assessment measures, such as the traditional Mehran score (Mehran et al., 2004) and ACEF scores (Andò et al., 2013), were originally designed for cardiac interventions and have not been validated for predicting the risk of CA-AKI in patients receiving intravenous contrast (Silver et al., 2015). Additionally, due to the low awareness of kidney disease among the representative sample of Chinese adults (Zhang et al., 2012), the number of participants at risk for CA-AKI was limited, with only 10.5% of CKD patients aware of their condition. Furthermore, outpatients are highly mobile and CA-AKI usually occurs within 48 h, making it difficult to detect in most cases. Unlike anaphylaxis, which can be easily identified, potential renal toxicity is often overlooked by patients even clinicians and radiologists.

Thyroid dysfunction (TD) is a rare but possible side effect of exposure ICM in the general population. Since ICM contains supraphysiological quantities of iodine, it may cause either hyperthyroidism (Hyper) or hypothyroidism (Hypo) (Bednarczuk et al., 2021). According to Kornelius et al. (2015), one in every 250 patients may be affected by ICM-induced Hyper or Hypo, leading to harmful consequences. These findings are supported by both single-institution retrospective analyses and a recent meta-analysis (Bervini et al., 2021). Clinicians must remain vigilant about potential thyroid TD caused by ICM, as many radiological exams employing ICM are routinely performed. Identifying undiagnosed TD before ICM exposure is crucial. Undiagnosed Hyper may be surprisingly prevalent in communities with an iodine deficit. In our study, two patients with untreated or uncontrolled Hyper had delayed CECT scanning. One patient was newly diagnosed with Hyper based on physical findings and serum thyroid hormone assays. The other patient had self-discontinued anti-thyroid medication for 2 weeks resulting in abnormal thyroid function. Both were referred to endocrinologists and treated with methimazole and beta-blockers. When assessing the potential risk of ICM-induced TD, clinical symptoms of TD, pre-existing thyroid diseases, and iodine intake should be considered.

### 4.2 Study strengths and limitations

As far as we know, this is the first management project led by clinical pharmacists on ICM infusion risk assessment. The involvement of pharmacists in patient care and multidisciplinary decision-making is essential. Clinical pharmacists have a pivotal role in detecting and evaluating ADRs (Salazar et al., 2022). In fact, a recent systematic review has shown that pharmacist-led interventions can significantly reduce the incidence of adverse drug events (Ali and Salahudeen, 2021). Given their specialized medication knowledge, training, and unique position at the interface between patients and medications, pharmacists are well-equipped to solicit information from patients more accurately and comprehensively than doctors and nurses. They can also obtain a patient’s history of drug allergies and medication use, identify drugs associated with nephrotoxicity, and provide individualized prophylactic medication regimens to prevent adverse reactions to ICM. With clinical pharmacists added to the multidisciplinary collaborative team, patients can receive clearer and more comprehensive advice from healthcare professionals.

Compared to previous studies, our study has taken into account all possible risks and conducted a comprehensive, individualized risk assessment for patients throughout the entire process of intravascular contrast media infusion during radiological examinations. Our risk management model is highly practical and suitable for use in all hospitals, as it is based on standardized work procedures, a stable workflow, comprehensive risk assessment, and specific implementation measures. No unique or cutting-edge technology is required, and the multidisciplinary team members can be trained to competently carry out this work. We have provided sufficient detail in this paper regarding the risk assessment workflow, intervention components, roles and responsibilities of team members, making replication of this model possible. So, this project can be implemented at the Grade II and III hospital in China.

It is important to consider several limitations when interpreting the findings of this study. Firstly, the study was conducted at a single center and had a relatively small sample size. The main focus of the study was to establish a standard working mode and process, and to objectively describe the results of risk assessment, rather than to conduct a comparative analysis before and after implementing risk management. Furthermore, patients with risk factors were identified through a self-assessment form, which may have resulted in some patients not participating in the assessment due to difficulties in understanding the information or different levels of emphasis on the risks associated with ICM infusion. Additionally, it may have been challenging to provide the risk assessment service to all patients, but high-risk patients may have been prioritized for its benefits. Despite these limitations, the study provides valuable insights and serves as a reference for improving risk assessment practices in the future. Conducting a pragmatic study with a larger population would be reasonable to further investigate this topic.

## 5 Conclusion

We have successfully implemented a comprehensive ICM infusion risk management process with high clinical practicability. We have clarified the roles and responsibilities of multidisciplinary team members in risk management, and ensured the professionalism and accuracy of risk assessment results. This has allowed us to timely identify, provide early warning, and control risk factors. It demonstrates the positive impact of a medication risk management service provided by a clinical pharmacist. This strategy can be replicated at numerous medical facilities.

## Data Availability

The original contributions presented in the study are included in the article/Supplementary Material, further inquiries can be directed to the corresponding authors.

## References

[B1] ACR Committee on Drugs and Contrast Media (2022). ACR comittee on drugs and contrast media. Virginia, United States: ACR Manual on Contrast Media.

[B2] Ali S. Salahudeen M. S. Bereznicki L. R. E. Curtain C. M. (2021). Pharmacist-led interventions to reduce adverse drug events in older people living in residential aged care facilities: A systematic review. A Syst. Rev. 87 (10), 3672–3689. 10.1111/bcp.14824 33880786

[B3] Andò G. Morabito G. de Gregorio C. Trio O. Saporito F. Oreto G. (2013). The ACEF score as predictor of acute kidney injury in patients undergoing primary percutaneous coronary intervention. Int. J. Cardiol. 168 (4), 4386–4387. 10.1016/j.ijcard.2013.05.049 23711447

[B4] Bednarczuk T. Brix T. H. Schima W. Zettinig G. Kahaly G. J. (2021). 2021 European thyroid association guidelines for the management of iodine-based contrast media-induced thyroid dysfunction. Eur. Thyroid. J. 10 (4), 269–284. 10.1159/000517175 34395299 PMC8314764

[B5] Bervini S. Trelle S. Kopp P. Stettler C. Trepp R. (2021). Prevalence of iodine-induced hyperthyroidism after administration of iodinated contrast during radiographic procedures: A systematic review and meta-analysis of the literature. Thyroid 31 (7), 1020–1029. 10.1089/thy.2020.0459 33327840

[B6] Böhm I. Morelli J. Nairz K. Silva Hasembank Keller P. Heverhagen J. T. (2017). Myths and misconceptions concerning contrast media-induced anaphylaxis: A narrative review. Postgrad. Med. 129 (2), 259–266. 10.1080/00325481.2017.1282296 28085538

[B7] Chen Q. L. Zhao X. Y. Wang X. M. Lv N. Zhu L. L. Xu H. M. (2017). Retrospective analysis of non-laboratory-based adverse drug reactions induced by intravenous radiocontrast agents in a Joint Commission International-accredited academic medical center hospital in China. Ther. Clin. Risk Manag. 13, 565–573. 10.2147/tcrm.s134265 28490883 PMC5414727

[B8] Chen Y. Wang H. Wan Z. Xu Y. Huo Y. Ge J. (2014). Effects of plant biomass on denitrifying genes in subsurface-flow constructed wetlands. Chin. J. Interventional Cardiol. 22 (6), 341–345. 10.1016/j.biortech.2014.01.137 24565872

[B9] Choukroun C. Leguelinel-Blache G. Roux-Marson C. Jamet C. Martin-Allier A. Kinowski J. M. (2021). Impact of a pharmacist and geriatrician medication review on drug-related problems in older outpatients with cancer. J. Geriatr. Oncol. 12 (1), 57–63. 10.1016/j.jgo.2020.07.010 32800700

[B10] Davenport M. S. Cohan R. H. Caoili E. M. Ellis J. H. (2009). Repeat contrast medium reactions in premedicated patients: Frequency and severity. Radiology 253 (2), 372–379. 10.1148/radiol.2532090465 19789241

[B11] Davenport M. S. Perazella M. A. Yee J. Dillman J. R. Fine D. McDonald R. J. (2020). Use of intravenous iodinated contrast media in patients with kidney disease: Consensus statements from the American College of radiology and the national kidney foundation. Radiology 294 (3), 660–668. 10.1148/radiol.2019192094 31961246

[B12] Davenport M. S. Perazella M. A. Yee J. Dillman J. R. Fine D. McDonald R. J. (2020). Use of intravenous iodinated contrast media in patients with kidney disease: Consensus statements from the American College of radiology and the national kidney foundation. Kidney Med. 2 (1), 85–93. 10.1016/j.xkme.2020.01.001 33015613 PMC7525144

[B13] Doña I. Bogas G. Salas M. Testera A. Moreno E. Laguna J. J. (2020). Hypersensitivity reactions to multiple iodinated contrast media. Front. Pharmacol. 11, 575437. 10.3389/fphar.2020.575437 33071787 PMC7538657

[B14] Everson M. Sukcharoen K. Milner Q. (2020). Contrast-associated acute kidney injury. BJA Educ. 20 (12), 417–423. 10.1016/j.bjae.2020.07.006 33456926 PMC7808023

[B15] John A. M. Yadav S. (2019). Effect of bolus administration of non-ionic radiopaque contrast media on blood pressure variation. Radiography 25 (4), 346–348. 10.1016/j.radi.2019.04.008 31582243

[B16] Kooiman J. Pasha S. M. Zondag W. Sijpkens Y. W. van der Molen A. J. Huisman M. V. (2012). Meta-analysis: Serum creatinine changes following contrast enhanced CT imaging. Eur. J. Radiol. 81 (10), 2554–2561. 10.1016/j.ejrad.2011.11.020 22177326

[B17] Kornelius E. Chiou J. Y. Yang Y. S. Peng C. H. Lai Y. R. Huang C. N. (2015). Iodinated contrast media increased the risk of thyroid dysfunction: A 6-year retrospective cohort study. J. Clin. Endocrinol. Metab. 100 (9), 3372–3379. 10.1210/jc.2015-2329 26168278

[B18] Li X. Liu H. Zhao L. Liu J. Cai L. Liu L. (2016). Clinical observation of adverse drug reactions to nonionic iodinated contrast media in population with underlying diseases and risk factors. Br. J. Radiol. 90 (1070), 20160729. 10.1259/bjr.20160729 27928926 PMC5685114

[B19] Li X. Zheng S. Qu M. (2018). Expert consensus on the safety of iodine contrast agent infusion in imaging department. J. Interventional Radiology 27 (8), 707–712. 10.3969/j.issn.1008-794X.2018.08.001

[B20] Loh W. Tang M. L. (2018). The epidemiology of food allergy in the global context. Int. J. Environ. Res. public health 15 (9), 2043. 10.3390/ijerph15092043 30231558 PMC6163515

[B21] McDonald J. S. Larson N. B. Kolbe A. B. Hunt C. H. Schmitz J. J. Maddox D. E. (2021). Prevention of allergic-like reactions at repeat CT: Steroid pretreatment versus contrast material substitution. Radiology 301 (1), 133–140. 10.1148/radiol.2021210490 34342504

[B22] Mehran R. Aymong E. D. Nikolsky E. Lasic Z. Iakovou I. Fahy M. (2004). A simple risk score for prediction of contrast-induced nephropathy after percutaneous coronary intervention: Development and initial validation. J. Am. Coll. Cardiol. 44 (7), 1393–1399. 10.1016/j.jacc.2004.06.068 15464318

[B23] Meth M. J. Maibach H. I. (2006). Current understanding of contrast media reactions and implications for clinical management. Drug Saf. 29 (2), 133–141. 10.2165/00002018-200629020-00003 16454540

[B24] Morcos S. K. (2005). Review article: Acute serious and fatal reactions to contrast media: Our current understanding. Br. J. Radiol. 78 (932), 686–693. 10.1259/bjr/26301414 16046418

[B25] Nakano S. Tsushima Y. Taketomi-Takahashi A. Higuchi T. Amanuma M. Oriuchi N. (2011). Hypertensive crisis due to contrast-enhanced computed tomography in a patient with malignant pheochromocytoma. Jpn. J. Radiol. 29 (6), 449–451. 10.1007/s11604-011-0573-y 21786102

[B26] Nash K. Hafeez A. Hou S. (2002). Hospital-acquired renal insufficiency. Am. J. Kidney Dis. 39 (5), 930–936. 10.1053/ajkd.2002.32766 11979336

[B27] Newmark J. L. Mehra A. Singla A. K. (2012). Radiocontrast media allergic reactions and interventional pain practice-a review. Pain Physician 15 (5), E665–E675. 10.36076/ppj.2012/15/e665 22996860

[B28] Nyman U. Ahlkvist J. Aspelin P. Brismar T. Frid A. Hellström M. (2018). Preventing contrast medium-induced acute kidney injury: Side-by-side comparison of Swedish-ESUR guidelines. Eur. Radiol. 28 (12), 5384–5395. 10.1007/s00330-018-5678-6 30132106

[B29] Rosado Ingelmo A. Doña Diaz I. Cabañas Moreno R. Moya Quesada M. C. García-Avilés C. García Nuñez I. (2016). Clinical practice guidelines for diagnosis and management of hypersensitivity reactions to contrast media. J. Investig. Allergol. Clin. Immunol. 26 (3), 144–155. 10.18176/jiaci.0058 27326981

[B30] Salazar A. Amato M. G. Shah S. N. Khazen M. Aminmozaffari S. Klinger E. V. (2022). Pharmacists’ role in detection and evaluation of adverse drug reactions: Developing proactive systems for pharmacosurveillance. Am. J. Health-System Pharm. 80 (4), 207–214. 10.1093/ajhp/zxac325 36331446

[B31] Schönenberger E. Martus P. Bosserdt M. Zimmermann E. Tauber R. Laule M. (2019). Kidney injury after intravenous versus intra-arterial contrast agent in patients suspected of having coronary artery disease: A randomized trial. Artery Dis. A Randomized Trial 292 (3), 664–672. 10.1148/radiol.2019182220 31264950

[B32] Schopp J. G. Iyer R. S. Wang C. L. Petscavage J. M. Paladin A. M. Bush W. H. (2013). Allergic reactions to iodinated contrast media: Premedication considerations for patients at risk. Emerg. Radiol. 20 (4), 299–306. 10.1007/s10140-012-1081-9 23430296

[B33] Schrijvers R. Demoly P. Chiriac A. M. (2019). Premedication for iodinated contrast media induced immediate hypersensitivity reactions. Curr. Treat. Options Allergy 6 (4), 538–553. 10.1007/s40521-019-00224-z

[B34] Sebastià C. Páez-Carpio A. Guillen E. Pao B. Garcia-Cinca D. Poch E. (2021). Oral hydration compared to intravenous hydration in the prevention of post-contrast acute kidney injury in patients with chronic kidney disease stage IIIb: A phase III non-inferiority study (nicir study). Eur. J. Radiology 136, 109509. 10.1016/j.ejrad.2020.109509 33516141

[B35] Shrestha S. Kc B. Blebil A. Q. Teoh S. L. (2022). Pharmacist involvement in cancer pain management: A systematic review and meta-analysis. J. Pain 23 (7), 1123–1142. 10.1016/j.jpain.2022.02.002 35151871

[B36] Silver S. A. Shah P. M. Chertow G. M. Harel S. Wald R. Harel Z. (2015). Risk prediction models for contrast induced nephropathy: Systematic review. Bmj 351, h4395. 10.1136/bmj.h4395 26316642 PMC4784870

[B37] Somashekar D. K. Davenport M. S. Cohan R. H. Dillman J. R. Ellis J. H. (2013). Effect of intravenous low-osmolality iodinated contrast media on patients with myasthenia gravis. Radiology 267 (3), 727–734. 10.1148/radiol.12121508 23360741

[B38] Sousa-Pinto B. Fonseca J. A. Gomes E. R. (2017). Frequency of self-reported drug allergy: A systematic review and meta-analysis with meta-regression. Ann. Allergy Asthma Immunol. 119 (4), 362–373. 10.1016/j.anai.2017.07.009 28779998

[B39] Topaz G. Karas A. Kassem N. Kitay-Cohen Y. Pereg D. Shilo L. (2018). Iodinated contrast media allergy in patients hospitalized for investigation of chest pain. J. Allergy Clin. Immunol. Pract. 6 (6), 2059–2064. 10.1016/j.jaip.2018.03.012 29655771

[B40] van der Molen A. J. Reimer P. Dekkers I. A. Bongartz G. Bellin M. F. Bertolotto M. (2018). Post-contrast acute kidney injury - Part 1: Definition, clinical features, incidence, role of contrast medium and risk factors: Recommendations for updated ESUR Contrast Medium Safety Committee guidelines. Eur. Radiol. 28 (7), 2845–2855. 10.1007/s00330-017-5246-5 29426991 PMC5986826

[B41] Zhang L. Wang F. Wang L. Wang W. Liu B. Liu J. (2012). Prevalence of chronic kidney disease in China: A cross-sectional survey. Lancet 379 (9818), 815–822. 10.1016/s0140-6736(12)60033-6 22386035

